# Differential susceptibility of mitochondrial complex II to inhibition by oxaloacetate in brain and heart

**DOI:** 10.1016/j.bbabio.2016.06.002

**Published:** 2016-09

**Authors:** Anna Stepanova, Yevgeniya Shurubor, Federica Valsecchi, Giovanni Manfredi, Alexander Galkin

**Affiliations:** a Queen's University Belfast, School of Biological Sciences, Medical Biology Centre, 97 Lisburn Road, Belfast BT9 7BL, UK; b N.K. Koltzov Institute of Developmental Biology, Russian Academy of Sciences, 26 Vavilova Str., Moscow 119334, Russia; c Weill Medical College of Cornell University, Brain and Mind Research Institute, Department of Neurology, 525 East 68th Street, Room A501, New York, NY 10021, USA; d Feil Family Brain and Mind Research Institute, Weill Cornell Medicine, 407 East 61st Street, New York, NY 10065, USA

**Keywords:** A/D, active/de-active transition, DDM, *n*-Dodecyl β-d-maltoside, FAD, flavin adenine dinucleotide, GOT1, glutamic/oxaloacetate transaminase, Type 1, HAR, hexaammineruthenium, I/R, ischemia/reperfusion, OAA, oxaloacetate, Q_1_, 2,3-dimethoxy-5-methyl-6-(3-methyl-2-butenyl)-1,4-benzoquinone, RET, reverse electron transfer, SDH, succinate dehydrogenase, SET medium, sucrose/EDTA/Tris medium, SMP, submitochondrial particles, TCA, tricarboxylic acid cycle, Mitochondrial complex II, Succinate dehydrogenase, Oxaloacetate, Ischemia, Krebs cycle

## Abstract

Mitochondrial Complex II is a key mitochondrial enzyme connecting the tricarboxylic acid (TCA) cycle and the electron transport chain. Studies of complex II are clinically important since new roles for this enzyme have recently emerged in cell signalling, cancer biology, immune response and neurodegeneration. Oxaloacetate (OAA) is an intermediate of the TCA cycle and at the same time is an inhibitor of complex II with high affinity (*K*_d_ ~ 10^− 8^ M). Whether or not OAA inhibition of complex II is a physiologically relevant process is a significant, but still controversial topic. We found that complex II from mouse heart and brain tissue has similar affinity to OAA and that only a fraction of the enzyme in isolated mitochondrial membranes (30.2 ± 6.0% and 56.4 ± 5.6% in the heart and brain, respectively) is in the free, active form. Since OAA could bind to complex II during isolation, we established a novel approach to deplete OAA in the homogenates at the early stages of isolation. In heart, this treatment significantly increased the fraction of free enzyme, indicating that OAA binds to complex II during isolation. In brain the OAA-depleting system did not significantly change the amount of free enzyme, indicating that a large fraction of complex II is already in the OAA-bound inactive form. Furthermore, short-term ischemia resulted in a dramatic decline of OAA in tissues, but it did not change the amount of free complex II. Our data show that in brain OAA is an endogenous effector of complex II, potentially capable of modulating the activity of the enzyme.

## Highlights

• Complex II in mitochondrial membranes is inhibited by tightly-bound oxaloacetate.
• Oxaloacetate binds to the heart enzyme during isolation.
• In brain a large fraction of Complex II is present in oxaloacetate-bound form.
• Short-time tissue ischemia does not affect the content of the free Complex II in brain.

## 1 Introduction

Mitochondrial complex II (EC 1.3.5.1, succinate:quinone reductase (SQR), succinate dehydrogenase (SDH)) catalyzes oxidation of matrix succinate to fumarate by membrane bound ubiquinone, thereby feeding electrons to the cytochrome *bc*_1_ complex. Complex II is composed of four nuclear encoded subunits. The structure of complex II resolved at 2.4 Å resolution [1] revealed that the hydrophobic subunits SDHC and SDHD are anchored to the inner mitochondrial membrane with a short segment extended into the intermembrane space, while the catalytic subunits SDHA and SDHB project into the matrix. Electrons are transferred from succinate to ubiquinone through the covalently bound flavin-adenine dinucleotide (FAD) and three iron-sulphur clusters [2]. SDHA is the largest subunit and contains a covalently attached flavin in the dicarboxylate-binding site, where succinate is oxidized to fumarate. The regulation of complex II activity is not well understood, which is surprising since this enzyme integrates the tricarboxylic acid (TCA) cycle metabolism with the mitochondrial respiratory chain.

As found in pioneering work of Singer [3], preparations of the enzyme isolated from tissues show a spontaneous increase in the rate of catalytic reaction. It was found that preparations of isolated SDH or enzyme in the membrane of submitochondrial particles (SMP) could be significantly activated by succinate and its analogues [4], [5], [6], [7]. This activation was attributed to a slow dissociation of the competitive inhibitor oxaloacetate (OAA) from the active centre of the enzyme. However, whether the observed inhibition of SDH by OAA is significant for physiological regulation of the enzyme *in vivo*, or just an artefact caused by the isolation procedure is still a matter of controversy [6], [8], [9]. Matrix OAA might be a potent physiological effector of SDH with a very high affinity to the dicarboxylate binding site of the enzyme (K_d_ ~ 10^− 8^ M) [7] or, it may bind to SDH during the isolation procedure due to the very fast rate of binding, and stay in a bound state even after repetitive washing or gel filtration [5], [10].

Studies of mitochondrial complex II are clinically important [11], since this enzyme has recently emerged as focal point of investigation in cell signalling [12], [13], [14], [15], cancer biology [16], [17], [18], [19], immunology [20], neurodegeneration [21] and cardiovascular conditions [22], [23]. Acute tissue ischemia is known to be associated with blockade of respiration, accumulation of reduced equivalents in the matrix (NAD(P)H), depletion of cellular ATP/phosphocreatine pool, and, eventually, impairment of mitochondrial function [24]. Hypoxia-dependent inhibition of complex I and II would have a strong effect on TCA cycle reactions [25]. In turn, this would result in significant alterations in the concentration of intermediates of the TCA cycle [22], [26], [27], including OAA and succinate. Therefore, it is crucial to know whether binding of OAA to SDH is a physiologically relevant process.

The aim of the present study was to investigate OAA inhibition of SDH in heart and brain and the effect of short-term global ischemia. Mitochondrial membranes were rapidly isolated from tissue samples, SDH activity was assessed and degree of OAA inhibition determined. We found that a significant fraction of SDH isolated from heart and brain cortex (30.2 ± 6.0% and 56.4 ± 5.6%, respectively) is in inactive form. Using an OAA-depleting system during isolation, we confirmed that OAA inhibition of the enzyme physiologically takes place *in situ* in the brain, but not in the heart. We also determined that short-term ischemia did not significantly change the fraction of free enzyme in both tissues.

## 2 Materials and methods

### 2.1 Experimental animals

Male C57BL/6J mice (10–12 weeks) were used for all studies. Mice were housed under constant climatic conditions with free access to food and water, except that they were fasted for 18 h prior to tissue harvesting. All experiments were performed in accordance with the Guidance on the Operation of the Animals (Scientific Procedures) Act, 1986 (UK).

Cardiac and respiratory arrest was initiated by cervical dislocation and carcasses for ischemic group were placed in a 37 °C incubator to maintain physiological body temperature. Either immediately (control group) or 15 min after cardiac arrest (ischemic group) animals were decapitated directly into liquid nitrogen. Hearts were rapidly extracted (within 30 s) and snap-frozen in liquid nitrogen immediately or 15 min after cardiac arrest.

### 2.2 Mitochondrial fraction isolation

The isolation of heart mitochondrial membranes was carried out essentially as described [28]. Particular care was taken to cool down all media, glassware, and centrifuge rotors (2–3 °C). Pieces of frozen heart of around 150 mg were placed into 2.5 ml of isolation medium (200 mM Tris-HCl, pH 8.5, 0.5 mM EDTA) and homogenized in an IKA tissue disruptor (Tissue Tearor, 985,370, 2 min, 6000 rpm). Tissue debris was discarded after brief centrifugation for 4 min at 1500*g*. The supernatant was centrifuged for 15 min at 20,000*g* and the membrane pellet was rinsed twice with SET medium (50 mM Tris-HCl (pH 7.5), 0.25 M sucrose, and 0.2 mM EDTA). Resulting brown mitochondria pellet was resuspended in 100 μl of the same buffer, aliquoted, frozen in liquid nitrogen and stored at − 80 °C until use.

Isolation of the brain mitochondrial membranes was performed as follows. Pieces of frozen forebrain (200 mg of tissue) were homogenized in 1 ml of MSE buffer (225 mM mannitol, 75 mM sucrose, 5 mM HEPES, 0.1% BSA, 1 mM EGTA, 0.1 mM EDTA pH 8.0) using 2 ml Kontes™ Dounce homogenizer, tight pestle, with 60–75 strokes. The next steps of isolation procedure were the same as described above for heart samples. The membrane pellet was rinsed with SET medium containing 0.1% BSA.

For isolation in the presence of an OAA-depleting system, homogenization buffers used for disruption of tissues were supplemented with 5 mM glutamate and glutamic/oxaloacetate transaminase Type 1 (GOT1) from porcine heart (Sigma G2751, 317 U/mg protein), 90 and 30 μg/ml for brain and heart, respectively.

### 2.3 Western blot

12 μg of protein were loaded on a 12% SDS-PAGE gel. Next, proteins were transferred electrophoretically to a PVDF membrane (EMD Millipore) in transfer buffer. After blotting, the membrane was blocked for 30 min with Odyssey blocking buffer (Li-Cor, diluted 1:1 in PBS). The blot was then incubated for 2 h at room temperature with either primary antibody against CII 70 kDa subunit (a11142, Molecular Probes, diluted 1:1000 in PBS + 0.01% Tween 20) or with total OXPHOS rodent primary western blot antibody cocktail (ab110413, Abcam, diluted 1:1000 in PBS + 0.01% Tween 20) containing 5 different antibodies against the 5 OXPHOS complexes: CI subunit NDUFB8, CII-30 kDa, CIII-Core protein 2, CIV subunit I and CV alpha subunit. The membrane was then washed 3 times for 10 min with PBS-Tween and incubated with secondary antibodies (IRDye680 conjugated goat anti-mouse IgG, Highly Cross Adsorbed (Li-Cor)), diluted 1:10,000 in Odyssey buffer and PBS-Tween. After 3 final washing steps of 10 min each, fluorescence scanning was performed using an Odyssey Imaging system (Li-Cor) and fluorograms were inverted for visualization purposes. In order to normalize the protein content, the blot was subsequently incubated overnight first with a primary antibody against TIM23 (BD Biosciences 1:1000) and then with a secondary antibody (IRDye680 conjugated goat anti-mouse IgG). Quantification of the bands was performed using the Image Studio software (Li-Cor).

### 2.4 TCA cycle intermediates determination

300μl of ice-cold 10% perchloric acid (PCA) was added to frozen brain or heart mouse tissue (20–30 mg) and briefly sonicated for 6–10 s on ice. The resulting homogenate was kept on ice for an additional 10 min. The precipitate was removed by a 10 min centrifugation at 14,000*g* in a pre-cooled centrifuge (4 °C), and the obtained supernatant was transferred into a new 1.5 ml tube for a second centrifugation in the same conditions. After the second centrifugation, the supernatant was used for direct injection into a HPLC system. The HPLC system included: 2489 Waters HPLC-UV/VIS detector set at 210 nm, Waters 1525 binary pump with an established flow rate of 0.45 ml/min, and Waters 2707 autosampler with pre-cooled platform. Organic acid separation was performed on a C18 reverse-phase analytical column (YMC, Triart, 250 × 3.0 mm I.D. particle size 3 μm, 12 nm), equipped with Phenomenex Security guard column (cartridge C18, 4 × 2 mm, PN# AJ0-4286). Both columns were maintained at room temperature. Organic acids were eluted with 20 mM KH_2_PO_4_ (pH 2.9) mobile phase. The chromatogram collection, storage and metabolite quantitation was performed using Breeze 2 software. All TCA cycle intermediates were identified and quantified per mg of wet tissue using the known standards. The peaks were routinely spiked with the standards to confirm their identities. Six to eight samples per each group were analysed. The analytical variability for OAA standard solution was established somewhere within 3–5% (n = 5). Analytical variability for OAA level in biological samples was slightly higher (somewhere within 10–15%).

### 2.5 Activity measurements

Oxidation of NADH was determined spectrophotometrically as a decrease in absorption at 340 nm (Perkin Elmer – Lambda 35, ε_340_ _nm_ = 6.22 mM^− 1^ × cm^− 1^) with 150 μM NADH in 1 ml of standard SET medium (pH = 7.5) supplemented with 10 μM cytochrome *c* and containing 10–25 μg protein/ml mitochondrial membranes. NADH:HAR oxidoreductase reductase [29] were assayed in the same medium supplemented with 1 mM cyanide and 1 mM HAR. NADH-oxidase was fully sensitive to complex I inhibitor rotenone.

For the determination of the ratio of the active and deactive form of mitochondrial complex I (A/D ratio) [30], [31] an alkaline buffer and 1 mM ferricyanide were used to allow rapid oxidation of reduced matrix pyridine nucleotides to prevent turnover-dependent complex I activation. This treatment is known to preserve the A/D ratio when membranes are isolated from tissues [28], [32]. Relative content of the A-form in a given preparation was measured as described previously [28], [31] using initial rates of NADH-oxidase reaction in alkaline (pH = 8.8) SET medium supplemented with 2 mM MgCl_2_ before and after activation with a pulse of 15 μM NADH.

Complex IV activity was measured spectrophotometrically as oxidation of 50 μM ferrocytochrome *c* at 550 nm (ε_550_ _nm_ = 21.0 mM^− 1^ × cm^− 1^) in the same medium supplemented with 0.025% DDM. Ferrocytochrome *c* oxidase activity was fully sensitive to cyanide.

After specific treatment of the membranes succinate-dependent activities were determined using two methods. First, succinate oxidase activity was measured using an Oroboros high-resolution respirometer in 2 ml of SET buffer containing 10 μM cytochrome *c* and 10 mM succinate. Second, succinate:ubiquinone reductase activity of SDH was determined spectrophotometrically at 275 nm (ε_275_ _nm_ = 13.0 mM^− 1^ × cm^− 1^, [33]) in SET medium with 15 μM Q_1_ as electron acceptor and 1 mM cyanide. The reaction was started by addition of 10 mM succinate. Both activities were fully sensitive to specific inhibitors such as malonate, OAA or thenoyltrifluoroacetone. Enzymatic activity with Q_2_ was somewhat higher, but due to the significant optical disturbance after addition of substrate to membranes in the activity assay, more hydrophilic Q_1_ was used in the final experiments.

For estimation of the fraction of OAA-free enzyme, initial rate of succinate-dependent activities were determined for membranes without any pretreatment. The total amount of enzyme was determined after malonate-activation of the preparation by preincubation with 1 mM malonate at 30 °C for 30 min, as already reported [34]. In such treatment malonate replaces OAA in the active centre of the enzyme. After dilution of the preparation in the activity buffer, the fast rate of malonate dissociation ensures rapid release of the inhibitor from the active centre of SDH, so that the full activity can be assessed. To account for any possible effect of temperature, membranes were incubated at 30 °C for 30 min. This treatment did not result in activation of the enzyme.

Activity of glutamic/oxaloacetate transaminase (GOT1) was measured spectrophotometrically, in 1 ml cuvette with constant stirring, as oxidation of 500 μM OAA (ε_256_ _nm_ = 0.356 mM^− 1^ × cm^− 1^, [35]) in the presence of 5 mM glutamate at 2–4 °C. No significant difference in activity was observed for both mediums used for heart or brain mitochondria membranes isolation.

All chemicals were purchased from Sigma. Protein content was determined by BCA assay (Sigma). All activities, except for GOT1, were measured at 25 °C. Data are presented as the arithmetic means ± S.D. and two-sample *t*-test was used to calculate *p* values. At least five animals were used per each group. The experimental details are described in the figure legends.

## 3 Results

### 3.1 Characterization of the preparations and effect of ischemia on respiratory chain enzymes

**Fig. 1:**
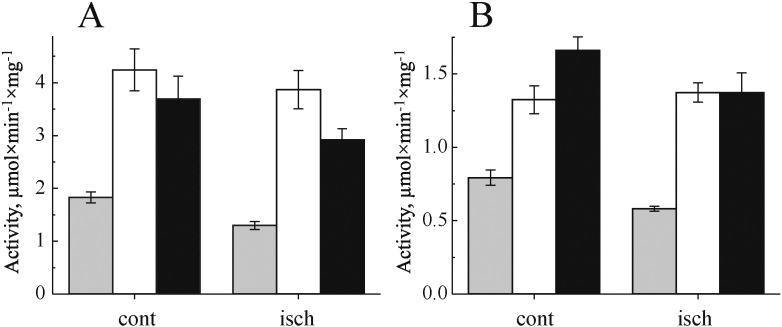
Effect of global ischemia on mitochondrial respiratory chain complexes activity in heart (A) and brain (B) membranes isolated from a control (cont) and ischemic (isch) tissue. NADH-oxidase (grey), NADH:HAR reductase (white) and cytochrome *c* oxidase (black) activities were determined spectrophotometrically as described in Materials and methods. n = 5.

Our isolation protocol was initially developed with the aim of fast isolation of mitochondrial membranes from heart and brain tissues. Catalytic properties of membrane preparations are summarized in Fig. 1. Oxidase activities were sensitive to classical inhibitors of respiration, such as cyanide. NADH-oxidase was 98% sensitive to rotenone. Neither the total NADH-oxidase (complexes I + III + IV) nor cytochrome *c* oxidase (complex IV) activities of mitochondrial membranes were significantly altered after ischemia, in heart and brain tissue (Fig. 1A and B). The relative content of complex I as measured by NADH:HAR reductase activity, which is proportional to the amount of enzyme in the membrane [36], [37], was also not affected by ischemia. It should be noted that, as expected, 15 min ischemia induced a significant shift in so-called reversible A/D transition of complex I [31] (around 18 and 6 fold increase in the content of the D-form in brain and heart, respectively (n = 4)).

**Fig. 2:**
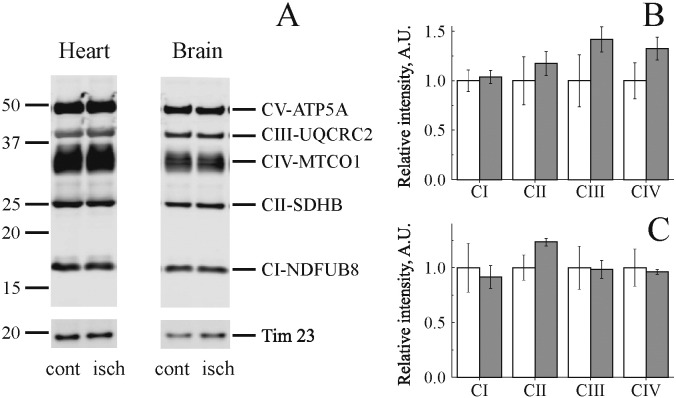
Effect of global ischemia on mitochondrial respiratory chain complexes content. (A) Representative Western blots of subunits of mitochondrial respiratory chain obtained from heart (left) and brain (right) mitochondrial membranes from control (cont) and ischemic (isch) tissues. Quantification of respiratory subunits abundance for heart (B) and brain (C) mitochondria. The intensity of all bands was normalized to the mitochondrial membrane protein Tim23, which showed no change in abundance in any group (white and grey bars show control and ischemic tissue, respectively). NDUFB8, complex I; SDHB succinate dehydrogenase subunits B; UQCRC2, *bc*_1_ complex; MTCO1, complex IV subunit 1; ATP5A, Complex V α-subunit. n = 3.

To exclude that ischemia had induced changes in the amount of mitochondrial membrane complexes we quantified their abundance by western blot. A representative western blot for subunits of the respiratory chain complexes using the Abcam OXPHOS antibody cocktail is shown in Fig. 2A. Ischemia did not significantly affect the abundance of mitochondrial complexes I–V subunits (Fig. 2B).

### 3.2 Global ischemia affects TCA cycle metabolites

**Fig. 3:**
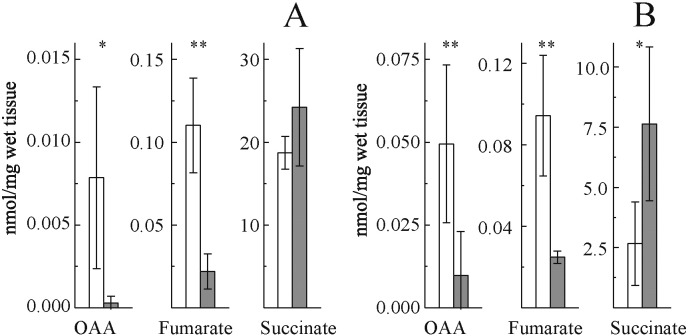
Effect of global ischemia on concentration of some TCA cycle metabolites in heart (A) and brain (B) tissue. White and grey bars correspond to control and ischemic samples, respectively. Determination was performed as described in Materials and methods. n = 6–8, *p < 0.04; **p < 0.01.

Ischemia results in accumulation of reducing equivalents (NAD(P)H) in the mitochondrial matrix and in a dramatic change in the concentration of TCA metabolites, possibly affecting the interaction of OAA with SDH. Therefore we assessed concentration of OAA, succinate, and fumarate in control and ischemic tissues (Fig. 3). A drastic drop of OAA content was observed in both heart and brain, with concomitant decrease of fumarate and rise of succinate.

### 3.3 Assessment of succinate-dependent activities of SDH

**Fig. 4:**
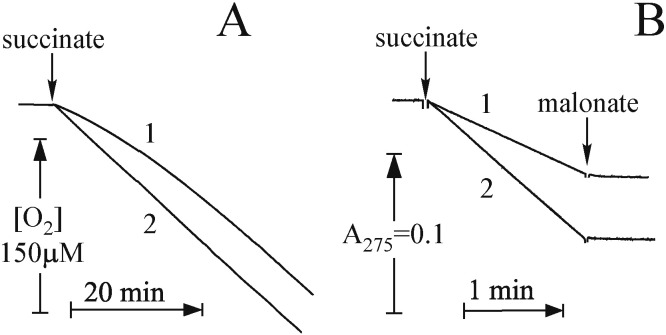
Time-course of succinate-dependent activities of heart mitochondrial membranes. Succinate oxidase and succinate:Q_1_ oxidoreductase were measured (A and B, respectively). Succinate oxidase reaction was started by addition of 20 mM succinate to a respirometer cell containing 12.5 μg/ml membranes in SET buffer supplemented with 10 μM cytochrome *c*. Succinate:Q_1_ reaction was started by the addition of 10 mM succinate to the spectrophotometer cuvette with assay mixture containing 5 μg/ml membranes and 15 μM Q_1_ in SET buffer in the presence of 1 mM cyanide. In A and B, traces 1 are from untreated membranes, while traces 2 are from malonate-pretreated membranes.

For the assessment of the state of SDH we measured succinate-oxidase activity (complexes II + III + IV) and reduction of the artificial acceptor ubiquinone-1 (Q_1_) by SDH in mitochondrial membranes. Representative traces for heart mitochondria are shown in Fig. 4. Both activities were fully sensitive to malonate (10 mM) or OAA (1 mM). Untreated heart mitochondrial membranes showed considerable lag phase during continuous assay of succinate oxidase reaction (Fig. 4A, trace 1), confirming previous observations with purified enzyme or SMP [4], [6], [7], [38]. This phenomenon indicated slow (half-time of around 20 min) dissociation of OAA from the active centre of SDH during the time of the assay. This lag-phase was completely eliminated by incubation of membranes with 1 mM malonate for 30 min at 30 °C prior to measurements. After this “activation” treatment the reaction rate became linear (Fig. 4A, trace 2). Contrary to succinate-oxidase, no lag was seen in our conditions, when reduction of Q_1_ was catalyzed by malonate-untreated preparation, due to the short time of the reductase assay (1–2 min). Activation with malonate significantly (> 50%) increased both succinate oxidase and quinone reductase activity in control samples, suggesting the displacement of tightly bound OAA from the active centre of the enzyme after incubation at 30 °C [7]. After dilution of the preparation in the mixture for activity measurement the fast rate of malonate dissociation ensures rapid release of the inhibitor from the active centre of SDH, so that the full activity can be assessed.

### 3.4 Inhibition of SDH by OAA

**Fig. 5:**
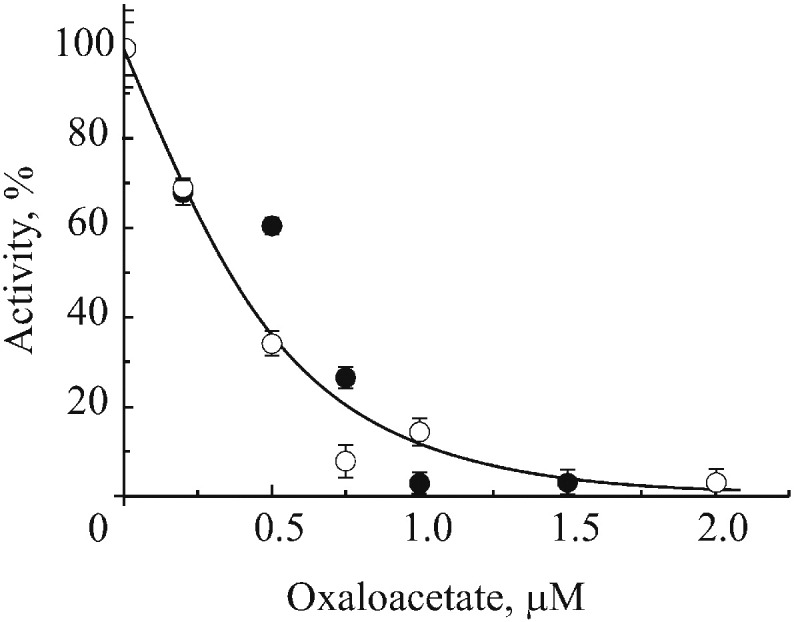
Inhibition of the succinate:Q_1_ reductase activity of heart and brain membranes by OAA (close and open circles, respectively). Before titration, membranes were resuspended to 5 mg/ml in SET buffer with 1 mM malonate for 30 min at 30 °C and briefly washed twice at 0 °C. Pellets were resuspended in the same buffer at concentration 1 mg/ml and OAA was added. After incubation for 1 h at room temperature succinate:Q_1_ activity was measured in SET buffer as described in Materials and methods. The activity without added OAA was 0.83 and 0.11 μmol Q_1_ × min^− 1^ × mg protein^− 1^ for heart (closed) and brain (open circles) membranes respectively.

Due to the reported tissue specific heterogeneity of SDH [39], [40], [41], we compared the sensitivity of the enzyme to OAA in heart and brain. Titration of succinate:Q_1_ reductase activity by OAA using mitochondrial membranes from brain and heart showed similar affinity of the inhibitor to the enzyme in both tissues (Fig. 5).

### 3.5 Effect of global ischemia on malonate activation of mitochondrial SDH

**Fig. 6:**
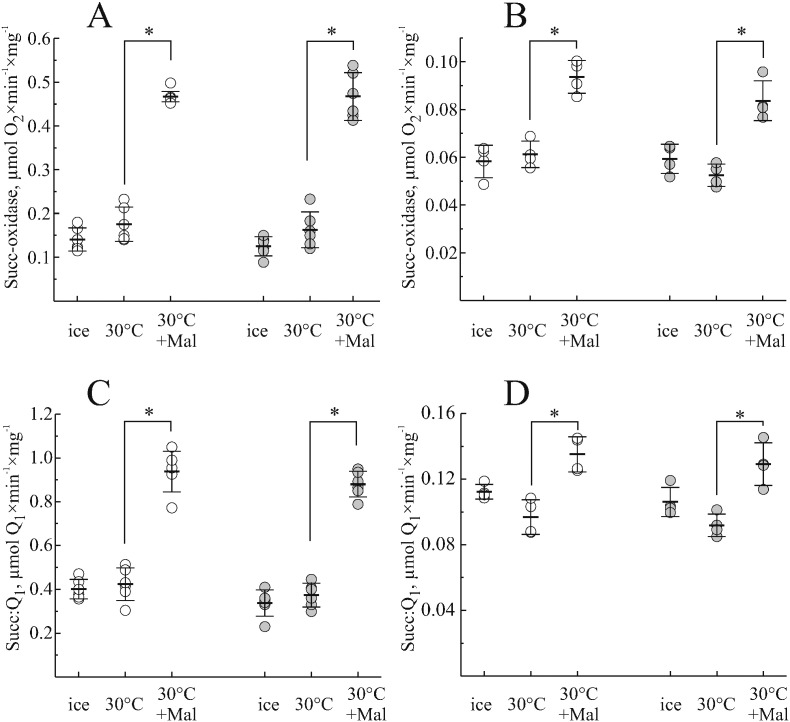
Effect of malonate activation on succinate oxidase (A, B) and succinate:Q_1_ reductase (C, D) activity of mitochondrial membranes isolated from heart (A, C) and brain (B, D) at 0 and 15 min after cardiac arrest (open and grey circles, respectively). Membranes were kept on ice or incubated with and without 1 mM malonate at 30 °C for 30 min. Activities were then assayed as described in Materials and methods. Here and in Fig. 7 each open circle represents data from a single mouse and horizontal lines depict the mean, and error bars indicate SD. *p < 0.005, n = 5.

To determine the effect of global ischaemia on OAA inhibition of SDH, mitochondrial membranes were isolated from brain and heart immediately (control) or 15 min after induction of global ischemia. Before activity measurements, the enzyme was activated by malonate and the activities of succinate oxidase (Fig. 6, A and B) or Q_1_-reductase (Fig. 6, C and D) were measured. Malonate incubation resulted in a significant activation of both activities of heart mitochondrial membranes (around 50%). Brain membranes were activated to the lesser extent (around 40%) indicating, that the fraction of free enzyme in brain was higher. We found that the degree of malonate activation in heart or brain was not different in the membranes obtained immediately or after 15 min ischemia.

### 3.6 Effect of OAA depletion during isolation on activation of SDH

**Fig. 7:**
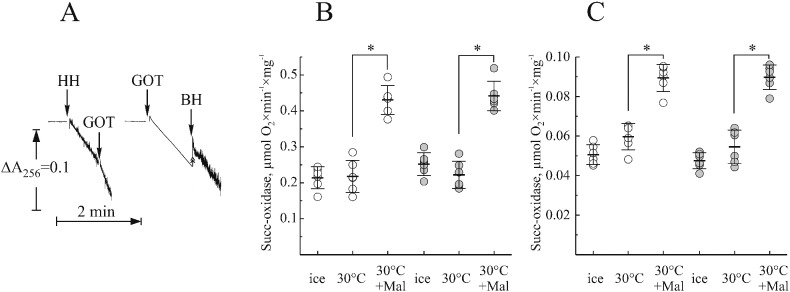
Malonate activation of succinate oxidase activity of mitochondrial membranes isolated in the presence of an OAA-depleting system. A, in the presence of 5 mM glutamate 3.75 μg/ml GOT1 effectively depletes OAA in tissue homogenisation medium. Addition of 1.5% heart or 0.5% brain homogenate (HH and BH, respectively) or GOT1 is shown by arrows. Heart (B) and brain (C) controls and 15 min after cardiac arrest (open and grey circles, respectively). Membranes were isolated in the presence of OAA-depleting system. Before kept on ice or incubated with or without 1 mM malonate for 30 min at 30 °C. Succinate oxidase activity was then assayed using Oroboros respirometer as described in Materials and methods. *p < 0.001, n = 5.

A clear effect of malonate activation on succinate-dependent activities of SDH, indicated that in both tissues only a small fraction of the enzyme is present in its free form. However the question is whether the presence of malonate-activatable SDH in the preparation reflects the situation *in situ* or if matrix OAA binds to the enzyme during the isolation. We developed a procedure for isolation of mitochondrial membranes in the presence of OAA-depleting system, using cytosolic glutamic oxaloacetate transaminase (GOT1). In the medium used for tissue homogenisation supplemented with glutamate, this enzyme catalyzed rapid transamination of OAA to aspartate (45 μmol OAA × min^− 1^ × mg^− 1^, Fig. 7A). At the same time, addition of 1.5% heart homogenate to the assay was effective in degrading added OAA (left trace), while addition of 0.5% brain homogenate did not significantly affect transamination of OAA by GOT1. Thus, the isolation medium was supplemented, during the initial tissue-disruption stage, with 30 or 90 μg/ml GOT1 for heart and brain, respectively. In the isolation conditions utilized (homogenization buffer, 2–3 °C, 5 mM glutamate), this amount of enzyme catalyzes the transaminase reaction with a rate of 25 or 75 μM/s. This was sufficient to deplete OAA in the homogenate (between 1 and 10 μM (from data in Fig. 3)) within the first second after tissue disruption.

Malonate activation of succinate oxidase of membranes obtained in the presence of GOT1 and glutamate during tissue homogenization was assessed (Fig. 7, B and C). In heart, this treatment significantly increased the fraction of free enzyme in control and ischemic samples (from 30.2 ± 6.0 to 49.8 ± 6.9%, *p* < 0.001 and from 27.1 ± 6.0 to 57.0 ± 4.4%, *p* < 0.001 for control and ischemic samples respectively). This indicated that OAA binds to the enzyme during isolation. On the contrary, in brain samples the presence of OAA-depleting system did not change the amount of free enzyme (56.4 ± 5.6 and 62.1 ± 3.4%, *p* = 0.12, for control samples). Therefore, we concluded that in brain *in situ* a significant fraction of the enzyme is already present in the inactive form and that OAA does not bind to SDH during isolation.

## 4 Discussion

Earlier investigations found that in preparations of SDH of various degree of purity, the enzyme was in a relatively inactivated state. Various treatments of the enzyme (ATP, reduced ubiquinone, TCA cycle metabolites and bromide ions) prior to activity measurement were used to obtain a higher, constant levels of activity [4], [5], [6], [42]. It was revealed that most of these treatments resulted in dissociation of OAA tightly bound in the active center and consequent activation of the SDH [4], [5], [6], [42]. If measured without any attempt to activate the enzyme by removal of OAA, progressive increase of the rate of succinate-oxidase reaction during onset of the assay can be observed. This activation is due to a slow dissociation of competitive inhibitor OAA from the active centre of the enzyme during continuous assay. OAA is a classical competitive inhibitor, with an extremely low dissociation rate (0.02 min^− 1^) [7], [43]. The lengths of the lag-phase depend on temperature, concentration of enzyme and substrate, as well as isolation protocol. Therefore, initial rates of the enzymatic reaction without activation do not reflect the full activity of the enzyme and could lead to underestimation of its real activity. In addition, alteration in concentration of TCA cycle intermediates in different conditions (*i.e.* normoxia/ischemia, wild type/mutant, the presence/absence of pharmacological agents) could affect the ratio between free and OAA-bound complex II in the preparation and complicate result interpretation.

The OAA content was lower in heart tissue than in brain confirming the pioneering studies [26], [44], [45], [46], [47], [48]. Most likely this is due to differences between metabolic pathways in neurones and cardiomyocytes, as well as to the heterogeneity of the brain tissue. It is difficult to determine whether the OAA level is high in neuronal or glial cells. Higher OAA may reflect greater levels of the TCA cycle anaplerotic pathways *via* combined activity of pyruvate carboxylase and transaminases responsible for biosynthesis of OAA in oligodendrocytes and astrocytes [49].

Acute ischemia is known to be associated with a depletion of tissue high-energy phosphates, increase in NADH/NAD^+^ ratio and alteration of TCA cycle intermediates [22], [26], [48]. We observed a dramatic drop of OAA and fumarate content with concomitant increase of succinate after 15 min ischemia in heart and brain tissues. It is in good agreement with previously published data [22], [26], [46], [48] and reversal of the malate dehydrogenase reaction due to the increased NADH/NAD^+^ ratio is probably the main cause of OAA decrease [27]. Possible mechanism of ischemic accumulation of succinate may involve reverse activity of SDH *via* fumarate reduction [22], [50], [51] as well as the conversion of succinyl-CoA to succinate [26], [27].

We analysed respiratory chain enzymes' activity of mitochondrial membranes obtained from brain and heart tissues in control and ischemic tissues (0 and 15 min after cardiac arrest). As expected, 15 min ischemia in both tissues did not significantly affect complex I or complex IV activity. In addition, no change in content of complex I (NADH:HAR reductase) was found. Activity of SDH was also similar in control and ischemic samples. We also assessed abundance of the respiratory chain complexes subunits, which was consistent with the activity measurements. These results indicate that 15 min ischemia does not significantly alter the content and activity of mitochondrial respiratory chain enzymes in heart and brain. Our data confirm earlier observations [28], [52], [53], [54] that lack of oxygen does not affect enzymes of the mitochondrial respiratory chain in the short term. At the same time, 15 min ischemia resulted in the conformational change (A to D transition) of mitochondrial complex I in both tissues, confirming results of our laboratory and others [28], [32], [55].

Two tissue-specific isoforms of the large subunit SDHA, bearing succinate-binding site, have been identified [39], [41]. In addition, SDHC transcript is subject to alternative splicing, resulting in three different isoforms, affecting enzyme activity [41]. Potentially, tissue-specific SDH isoforms could have different sensitivity to inhibitors and therefore we examined the effect of OAA on enzyme from brain and heart membranes. No difference in affinity of the brain and heart enzyme to OAA was observed, which is in close agreement with published data [7].

When succinate-dependent activities of heart and brain SDH were analysed, we found that the fraction of the free active enzyme was 35–40% in heart and 60–70% in brain. Short-term (15 min) ischemia did not change these proportions in either tissue. This finding is intriguing for two reasons. First, our data indicates that OAA content drops more than 5 and 10 fold during brain or heart ischemia respectively, while succinate rises, which is in agreement with published data [22], [27], [47], [48]. Second, affinity of OAA to the reduced enzyme is 10 fold lower than to oxidized [7]. Therefore, ischemia resulting in reduction of the respiratory chain should potentially have a strong effect on OAA binding to the enzyme. However, no significant difference between the degree of OAA inhibition in control and 15 min ischemia was found, in both tissues. This apparent discrepancy could be explained by the fact that OAA could bind to the enzyme during the isolation procedure since the rate constant of inhibitor binding to bovine enzyme is very high (*k*_*on*_ ~ 10^6^ M^− 1^ min^− 1^ [7], [43]) Thus, the fraction of the free enzyme in the preparation might not reflect the situation *in situ*.

Therefore, we implemented an OAA-depleting system with GOT1/glutamate at the homogenization step in order to quickly remove OAA from the homogenate. It should be stressed that this system in the implemented conditions was able to catalyze transaminase reaction with the rate of 25–75 μM/s, which is enough to process all available OAA in homogenate within first seconds (OAA content was taken as 0.0075 and 0.05 nmol/mg wet tissue for heart and brain respectively). Elimination of OAA at this stage did not change the fraction of free enzyme in brain, but significantly increased free SDH in heart.

Based on these results we concluded that in heart a fraction of the enzyme binds OAA during the preparation. This observation is in agreement with earlier publications where no OAA inhibition was found when heart mitochondrial membranes were isolated in the presence of malonate [8]. Malonate reversibly binds to SDH and prevents OAA inactivation of the enzyme in the homogenate, in the same way as the OAA-depleting system implemented in this study. Importantly, the data indicate that in brain OAA inhibition takes place *in situ,* suggesting that OAA could be an endogenous effector of SDH in brain. This is not unprecedented in other tissues, since SDH inhibition by OAA in liver mitochondria, resulting in reversible suppression of succinate oxidation, was observed during hibernation of ground squirrels [56]. Based on the OAA content in tissues, it is still difficult to assess the actual concentration of OAA in the matrix, where it potentially interacts with SDH. However, one reason for the difference between OAA effect on SDH in the brain and in the heart could that there is significantly more OAA in brain than in heart. In addition, greatly increased rate of binding of the OAA to the enzyme in Leigh syndrome associated with a point mutation in SDHA was thought to account for the severity of the disease, supporting idea of OAA regulating complex II [57].

Surprisingly, no effect of short-term ischemia on the content of the free enzyme was found. On the one hand, it could indicate that despite a sharp decrease in OAA tissue concentration, the inhibitor is still bound to the enzyme because of its very slow rate of dissociation (0.02 min^− 1^) [7], [43]. Therefore, 15 min ischemia may not be long enough to induce a significant increase in free enzyme content. Another possibility is that the inactivating OAA-induced conformational change in SDH may occur after the binding of the inhibitor, and the subsequent removal of OAA does not activate the enzyme [6], [58], [59]. Therefore, the state of SDH *in situ* is determined by the conformational change of the enzyme rather than OAA concentration in the matrix *per se*. Activating/inactivating conformational change phenomena have been observed in other enzymatic systems including mitochondrial complex I [31], [60], bacterial NiFe hydrogenases [61], methionine synthase [62], D-amino acid oxidase [63] and conformational change of complex II is currently being investigated in the authors' laboratory.

What could be a physiological role of OAA inhibition? SDH and complex I together potentially can catalyze so-called reverse electron transfer (RET) reaction, when succinate is used to reduce NAD^+^ at the expense of proton-motive force [60]. This process would be dependent on the catalytic rate of complex II. Inhibition of succinate oxidation by specific inhibitors (malonate, atpenin or thenoyltrifluoroacetone) was shown to decrease production of H_2_O_2_ by intact mitochondria [64], [65]. In intact mitochondria, rates of ROS production during RET are considerably higher than in any other conditions and it was suggested that this reaction could be responsible for generation of excess of ROS during ischemia/reperfusion [22]. Differential contribution of TCA cycle intermediates and the conditional removal of endogenous OAA are key factors that control succinate-dependent ROS generation during RET [11], [59], [66], [67]. Therefore, maintenance of a SDH fraction in the “inactive” form *in situ* may serve as a mechanism to modulate RET-dependent ROS-production [11].

The emerging role of SDH in cancer, neurological disorders [16], [17], [18], [19], [21], [57], [68], cell signalling [12], [13], [14], [15] (including stabilization of transcriptional factor HIF-1α [19]), immune response [20] and cardiovascular conditions [22], [23] highlights the need to determine if OAA has a regulatory function. Moreover, the use of a correct method to measure the activity of SDH that takes into account potential OAA inhibition is crucial. When measuring activity without the activation procedure, potential differences in enzyme state can be obscured or even reversed depending on the levels of OAA-bound and free enzyme. Therefore, adopting reliable methods for measuring SDH activity is crucial for biological research in these areas. In isolated mitochondrial membranes, complex II is only partially active. Thus, to measure total complex II activity it is essential to ensure that the enzyme is fully activated either by preincubation with succinate or malonate [7], [69].

In summary, the data presented here indicate that OAA inhibition of SDH occurs in brain, where OAA could be considered as a potential endogenous effector of SDH activity. Furthermore, relevant to other tissues, where OAA binds to SDH during isolation, such as heart, our findings are important for the methodology of SDH activity measurements.

## Transparency Document

Transparency document. Image 1
